# Association of elevated blood pressure and impaired vasorelaxation in experimental Sprague-Dawley rats fed with heated vegetable oil

**DOI:** 10.1186/1476-511X-9-66

**Published:** 2010-06-23

**Authors:** Xin-Fang Leong, Mohd Rais Mustafa, Srijit Das, Kamsiah Jaarin

**Affiliations:** 1 Department of Pharmacology, Faculty of Medicine, Universiti Kebangsaan Malaysia, Jalan Raja Muda Abdul Aziz, 50300 Kuala Lumpur, Malaysia; 2 Department of Pharmacology, Faculty of Medicine, University of Malaya, 50603 Kuala Lumpur, Malaysia; 3 Department of Anatomy, Faculty of Medicine, Universiti Kebangsaan Malaysia, Jalan Raja Muda Abdul Aziz, 50300 Kuala Lumpur, Malaysia

## Abstract

**Background:**

Poor control of blood pressure leads to hypertension which is a major risk factor for development of cardiovascular disease. The present study aimed to explore possible mechanisms of elevation in blood pressure following consumption of heated vegetable oil.

**Methods:**

Forty-two male Sprague-Dawley rats were equally divided into six groups: Group I (control) - normal rat chow, Group II - fresh soy oil, Group III - soy oil heated once, Group IV - soy oil heated twice, Group V - soy oil heated five times, Group VI - soy oil heated ten times. Blood pressure was measured at the baseline level and at a monthly interval for six months. Plasma nitric oxide, heme oxygenase and angiotensin-converting enzyme levels were measured prior to treatment, at month-three and month-six later. At the end of treatment, the rats were sacrificed and thoracic aortas were taken for measurement of vascular reactivity.

**Results:**

Blood pressure increased significantly (*p *< 0.01) in the repeatedly heated oil groups compared to the control and fresh soy oil groups. Consumption of diet containing repeatedly heated oil resulted higher plasma angiotensin-converting enzyme level and lower nitric oxide content and heme oxygenase concentration. Reheated soy oil groups exhibited attenuated relaxation in response to acetylcholine or sodium nitroprusside, and greater contraction to phenylephrine.

**Conclusion:**

As a result of consumption of repeatedly heated soy oil, an elevation in blood pressure was observed which may be due to the quantitative changes in endothelium dependent and independent factors including enzymes directly involved in the regulation of blood pressure.

## Introduction

Hypertension is one of the major risk factor of cardiovascular disease. This has contributed to be a leading cause of death in most developed and developing countries [1-4]. According to The Seventh Report of the Joint National Committee on Prevention, Detection, Evaluation and Treatment of High Blood Pressure, high blood pressure is defined as systolic blood pressure (SBP) which is greater than 140 mmHg and/or diastolic blood pressure (DBP) which is greater than 90 mmHg [5]. Patients in the pre-hypertensive stage, the SBP ranging between 120 mmHg and 139 mmHg, or DBP of 80 mmHg to 89 mmHg, are prone to develop hypertension and require medical attention [5].

It has been hypothesized that high blood pressure is associated with the imbalance between amount of antioxidants and reactive oxygen species (ROS) [6-8]. Endothelial dysfunction associated with abnormal endothelium-dependent relaxation is observed in hypertension [9,10]. This may be due to reduced nitric oxide (NO) bioavailability i.e. reduced in production or increased deactivation in the blood vessel wall [11,12]. The endothelium maintains vascular homeostasis by releasing NO, a regulatory substance which is known as an endothelium-dependent relaxing factor [13]. Production of toxic ROS such as superoxide anion may cause cellular damage by oxidizing nucleic acids, proteins and membrane lipids [14]. ROS may react with NO to form cytotoxic oxidant such as peroxynitrite which may compromise endothelial integrity [15,16].

Heme oxygenase (HO) is involved in the enzymatic conversion of pro-oxidative heme to iron, biliverdin and carbon monoxide. Biliverdin is subsequently being metabolized to antioxidant bilirubin [17]. In addition, carbon monoxide has numerous functions, such as relaxation of blood vessel and inhibition of platelet aggregation [18-20]. HO isoform 1 (HO-1) is inducible and sensitive to various stimuli that causes oxidative stress. As a result of these vasoactive properties, it is reasonable to suggest that HO-1 contributes to the regulation of blood pressure.

Previous observations have reported up-regulation of HO-1 in response to angiotensin (Ang) II *in vitro *and *in vivo *[21,22]. Ang II is produced by the catalyzation of angiotensin-converting enzyme (ACE) with angiotensinogen. Ang II induces oxidative stress with the activation of reduced form of nicotinamide adenine dinucleotide phosphate/nicotinamide adenine dinucleotide (NADPH/NADH) oxidase as well as generation of ROS [23,24]. Furthermore, Ang II increases lipid peroxidation and stimulates production of cytokines as well as adhesion molecules that act as pro-oxidants [25,26]. Chronically, unabated these reactions inevitably cause a rise in the blood pressure.

Frying is a process where heat and mass transfer as well as physical changes and chemical reactions take place [27]. Temperature and duration of heating, degree of oil saturation, presence of pro- and anti-oxidants, types of oil used are among the various factors affecting quality of dietary cooking oil. Several chemical reactions take place during the frying process such as hydrolysis, oxidation and polymerization [27]. These reactions alter the chemical structure of the oil molecules with the unsaturated fatty acids mostly changed.

In this modern fast paced society, fried food is gaining popularity in our daily diet. In fact, high oxidized fatty acid source is provided through consumption of these fried foods. Exposing cooking oil to deep-frying temperatures affects the chemical composition of fatty acids, with a configuration change from *cis *to *trans *isomers. Additionally, generation of oxidized products leads to a deleterious effect in the vascular function. Previous research findings in our laboratory have clearly shown that repeatedly heated palm oil causes a significant elevation in blood pressure [28,29]. It has been already documented that consumption of repeatedly heated palm oil increases blood pressure due to the alteration in endothelium-dependent vasorelaxant responses [29].

Soy oil (*Glycine max*), which is rich in polyunsaturated fatty acid was chosen for the present study as it was one of the most widely used edible oil throughout world. Often, the practice of reusing the oil in food preparation to reduce cost imposes deleterious effects on health. The current study was undertaken to observe the possible biochemical and vascular mechanisms involved in the increase of blood pressure following long term ingestion of heated soy oil in an experimental rat model.

## Materials and methods

### Animals and study design

Forty-two adult male Sprague-Dawley rats (aged 3 months), weighing 200 - 280 g were obtained from the Animal Source Unit, Universiti Kebangsaan Malaysia. The rats were randomly assigned into six dietary groups (one control and five experimental groups) comprising of seven animals each. Prior study approval was obtained from the University Research Secretariat and the University Animal Ethics Committee. All animal management and procedures were performed in accordance with the recommended guidelines.

The rats were kept in stainless-steel cages and maintained at room temperature of 27°C ± 2°C with a 12 h light-dark cycle. All rats had free access to food and water *ad libitum *during the study period. After one week of acclimatization, each group of rats were fed on the following diets: the group I (control) was fed only with commercial rat chow (basal diet); group II was fed with basal diet complement along with 15% weight/weight (w/w) of fresh soy oil (FSO); group III was fed with basal diet along with soy oil heated once (1HSO); group IV was fed with basal diet along with soy oil heated twice (2HSO); group V was fed with basal diet along with soy oil heated five times (5HSO) and group VI was fed with basal diet along with soy oil heated ten times (10HSO) for six months. Blood pressure was measured at baseline and at intervals of four weeks for 24 weeks using non-invasive method. Blood was collected through orbital sinus prior to treatment, at the week-12 and at the end of study. The blood was then centrifuged to obtain plasma and later stored at -70°C for further biochemical analyses. The animals were then sacrificed and thoracic aortas were isolated for measurement of vascular reactivity.

### Preparation of oil diet

Soy oil used in this study was purchased from a local source. It was used either in fresh form, heated once, twice, five times or ten times following method described by Owu *et al*. [30] with some modifications. Briefly, 2.5 L of oil was heated to 180°C in a stainless-steel wok and used to deep-fry 1 kg of peeled and sliced sweet potatoes. The heating process lasted for 10 min. The hot oil was then left to cool at room temperature for five hours. This would be the soy oil heated once (1HSO). The pre-cooled hot oil was used to deep-fry another new batch of sweet potatoes. This would be the soy oil heated twice (2HSO). The frying process was carried out with null replenishment of fresh oil. In order to obtain soy oil heated five times (5HSO) and soy oil heated ten times (10HSO), the same heating procedure was repeated four and nine times, respectively. The experimental diets were prepared weekly. Standard rat chow (Gold Coin, Port Klang, Selangor, Malaysia) was grinded and mixed with water and fresh or the heated soy oil prepared. The weight ratio of rat chow to the oil was 100:15. The mixture was then dried at 70°C overnight in an oven.

### Estimation of fatty acid composition

Fatty acid composition of fresh oil and oil subjected to different frying levels was analyzed using gas chromatography (GC-17A, Shimadzu, Kyoto, Japan) coupled with flame ionization detector (FID) and a BPX 70 capillary column (30 m × 0.25 mm × 0.25 μm). Oil sample of 0.1 ml was transesterified to fatty acid methyl esters using 1 ml of sodium methoxide (NaOMe 1 M) in 1 ml of hexane prior injection into the gas chromatography. Nitrogen was used as carrier gas in the analysis at a flow rate of 0.40 ml/min. The injector temperature was programmed at 250°C and the detector temperature was set to 280°C. Injection volume was 1 μl. Fatty acid methyl ester peaks were identified by comparing their retention times with authentic standards analyzed under the same condition. Fatty acid composition was expressed as percentage of the total fatty acids.

### Estimation of peroxide value

The peroxide value of oil was determined according to the American Oil Chemists' Society (AOCS) standard titration method (Official method Cd 8-53). Peroxide value was expressed as milliequivalents of active oxygen per kilogram of oil sample, mEq O_2_/kg.

### Measurement of blood pressure in rats

Systolic blood pressure of pre-warmed conscious rats was measured by the non-invasive tail cuff method using PowerLab data acquisition systems (ADInstruments, Castle Hill, NSW, Australia).

### Analysis of plasma nitric oxide (NO)

An earlier documented protocol was followed [29]. NO content was indirectly measured by its metabolite nitrite. Samples of 50 μl were taken in a microtiter plate and mixed with equal volumes of modified Griess reagent (Sigma-Aldrich, St. Louis, MO, USA). Incubation was continued for 15 min at room temperature in dark environment and the nitrite concentration was measured spectrophotometrically of the absorbance at 540 nm on Emax ELISA microplate reader using SoftMax Pro Software (Molecular Devices, Sunnyvale, CA, USA). Nitrite concentration was determined by performing standard curve with increasing concentration of sodium nitrite (Sigma-Aldrich, St. Louis, MO, USA).

### Aortic rings preparation and vascular reactivity

The aortic rings were prepared as per previous protocol [29] and described by Ajay and Mustafa [31]. The descending thoracic aorta was dissected and excess fat and connective tissues were removed. The aorta was cut into ring segments with the width of 3-5 mm. Aortic rings were suspended in 5 ml tissue baths containing Krebs physiological salt solution of the following composition (mM): NaCl 118.0, KCl 4.7, CaCl_2_·2H_2_O 2.5, KH_2_PO_4 _1.2, MgSO_4 _1.2, glucose 11.7, NaHCO_3 _25.0, and EDTA 0.026. The bathing solution was maintained at 37°C and continuously gassed with mixture of 95% oxygen and 5% carbon monoxide. Measurement of tissue isometric tension (g) was recorded by a force-displacement transducer (FT03E, Grass Instruments, West Warwick, RI, USA) attached to a MacLab recording system (MacLab model 8 S, ADInstruments, Castle Hill, NSW, Australia). The aortic rings were allowed to equilibrate for 30 to 45 min prior to the initiation of experimental protocol. The bathing solution was replaced every 15 min and resting tension was readjusted to basal tension 1 g whenever it is needed.

Following the equilibration period, the aortic rings were allowed to achieve maximal tension by exposure to stimulation of isotonic KCl solution (high K^+^, 80 mM). Following the washout of responses to high K^+^, the rings were constricted with phenylephrine (PE, 10^-7 ^M) to confirm the presence of the endothelium by the occurrence of relaxations induced by a single addition of acetylcholine (ACh 10^-5 ^M). Only the endothelial intact rings with more than 50% relaxation to ACh were used. All experiments were performed on different aortic rings with endothelium: (1) the cumulatively increasing concentration of relaxation responses to acetylcholine (ACh 10^-10 ^M to 10^-5 ^M) or sodium nitroprusside (SNP 10^-11 ^M to 10^-6 ^M) was recorded in phenylephrine (PE 10^-6 ^M) pre-contracted aortic rings. Dose-response curves were plotted as percentage of relaxation against the maximal PE (10^-6 ^M) contraction; (2) the contractile responses to cumulatively increasing concentration of PE (10^-10 ^M to 10^-5 ^M) were recorded in the rings and expressed as percentage of maximum contraction obtained with high K^+^.

### Drugs

The drugs chosen for this vascular reactivity study included acetylcholine chloride, phenylephrine-HCl (Sigma Chemical Co, St. Louis, MO, USA), sodium nitroprusside and Krebs salts (BDH Limited and BDH Laboratory Supplies, Poole, England) which were in fact used in an earlier work involving heated palm oil [29].

### Measurement of plasma heme oxygenase (HO) enzyme

Activity of HO-1 enzyme was determined enzymatically using commercially available kit (Assay Designs, Ann Arbor, MI, USA) following manufacturer's instruction. The intensity of coloured product was measured in a microplate reader (Molecular Devices, Sunnyvale, CA, USA) at 450 nm.

### Measurement of plasma angiotensin-converting enzyme (ACE)

The activity of ACE was measured using commercially available kit (USCNLife, West Lake, Wuhan, China) following manufacturer's instruction. The intensity of coloured product was measured in a microplate reader (Molecular Devices, Sunnyvale, CA, USA) at 450 nm.

### Data analysis

Results were reported as means ± S.E.M. unless otherwise stated. Normality of the data was determined using Kolmogorov-Smirnov test. Statistical differences were determined using paired student's *t *test, or one-way ANOVA followed by Tukey's HSD post-hoc test to identify the differences using SPSS version 13.0 (SPSS Inc, Chicago, IL, USA). Data which were not normally distributed were analyzed using Kruskal-Wallis *H *and Mann-Whitney *U *tests. Values of *p *< 0.05 were considered to be statistically significant.

## Results

### Fatty acid composition of oil

All the main constituents of fatty acid were present in the oil regardless of the number of times the soy oil was deep-fried (Table 1).

**Table 1:** Fatty acid composition and peroxide value of oils fed to rats.

	FSO	1HSO	2HSO	5HSO	10HSO
Fatty acid					
SFA (%)	16.69	17.14	18.32	18.10	18.39
MUFA (%)	25.0	26.10	27.39	24.21	23.43
PUFA (%)	52.48	51.78	50.14	41.72	43.19
Peroxide values # (mEq O_2_/kg)	4.84 ± 0.37^a^	5.35 ± 0.52^b^	10.31 ± 0.25^abc^	11.54 ± 0.29^abc^	12.52 ± 0.36^abc^

FSO, fresh soy oil; 1HSO, soy oil heated once; 2HSO, soy oil heated twice; 5HSO, soy oil heated five times; 10HSO, soy oil heated ten times; SFA, saturated fatty acids; MUFA, monounsaturated fatty acids; PUFA, polyunsaturated fatty acids. Same letters indicate significant difference between groups (*p *< 0.05). # Values are average of three estimations (means ± S.D.).

### Peroxide value of oil

Peroxide values showed a two-fold increment (*p *< 0.05) for 2HSO, and a three-fold increment for both 5HSO and 10HSO, compared to the fresh-oil value (Table 1).

### Body weight and food intake

The animals consumed the diet and grew well during the experimental study. The body weight in the control and test groups showed a significant increase (*p *< 0.05) at the end of the feeding period compared to their respective baseline values. However, there was no significant difference in body weight gain amongst the groups. Food intake was significantly decreased (*p *< 0.05) in groups fed heated soy oil (Table 2).

**Table 2:** Food intake and body weight gain in rats fed with respective soy oil diets.

Groups	Food intake (g/week)	Weight gain (g)
Control	172.5 ± 4.67	275.43 ± 29.13
FSO	152.7 ± 3.13	245.43 ± 13.90
1HSO	148.5 ± 3.25	252.86 ± 25.20
2HSO	140.3 ± 2.67*	212.71 ± 26.06
5HSO	136.4 ± 2.98*	230.14 ± 30.84
10HSO	132.9 ± 2.51*	209.71 ± 15.55

FSO, fresh soy oil; 1HSO, soy oil heated once; 2HSO, soy oil heated twice; 5HSO, soy oil heated five times; 10SO, soy oil heated ten times

* Significant difference (*p *< 0.05) compared to the control.

### Blood pressure

**Figure 1:**
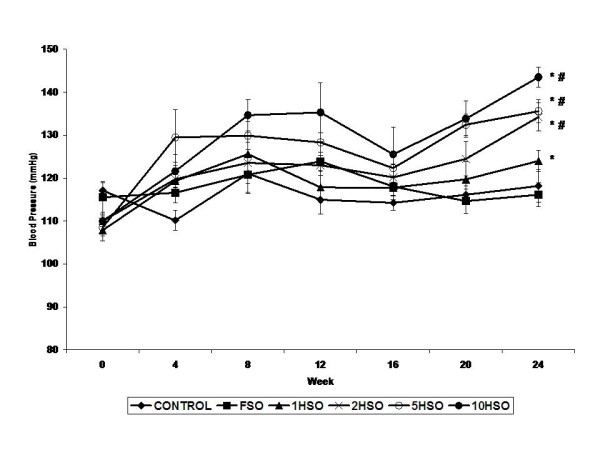
**Effects of fresh and heated soy oil on blood pressure in adult male rats**. Shown are the blood pressure changes in rats fed with basal diet (control), fresh soy oil (FSO), soy oil heated once (1HSO), soy oil heated twice (2HSO), soy oil heated five times (5HSO) or soy oil heated ten times (10HSO) after 24 weeks of feeding. Data are shown as means ± S.E.M. (n = 7), *p *< 0.01 indicates significant difference *between pre-and post-treatment values for the same group, #compared to control and FSO groups.

During the 24-week feeding periods, there was a significant increase (*p *< 0.01) in blood pressure in the groups fed with heated soy oil at the end of the study. Repeatedly heated oil groups consist of 2HSO, 5HSO and 10HSO had a significant difference (*p *< 0.01) compared to the control and FSO groups. On the other hand, the rats fed basal diet and FSO did not show any significant changes in blood pressure compared to their respective baseline values (Fig. 1).

### Nitric oxide (NO) metabolite level in plasma

**Figure 2:**
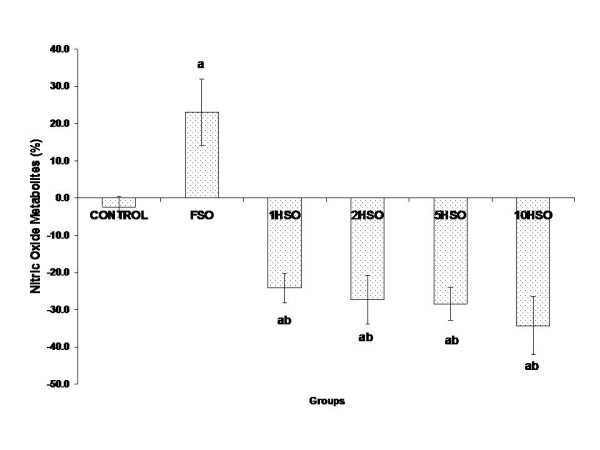
**Effects of fresh and heated soy oil on nitrite level in adult male rats**. Shown are the nitric level changes in rats fed with basal diet (control), fresh soy oil (FSO), soy oil heated once (1HSO), soy oil heated twice (2HSO), soy oil heated five times (5HSO) or soy oil heated ten times (10HSO). The results are expressed as percentage based on baseline values. Data shown as means ± S.E.M. (n = 7), *p *< 0.05 indicates significant difference compared to ^a^control, ^b^FSO group.

The FSO significantly increased (*p *< 0.05) NO metabolite level at the end of study. When rats were fed with heated oil, NO metabolite level in plasma was significantly reduced (*p *< 0.05) as shown in Fig. 2.

### Vascular response

**Figure 3:**
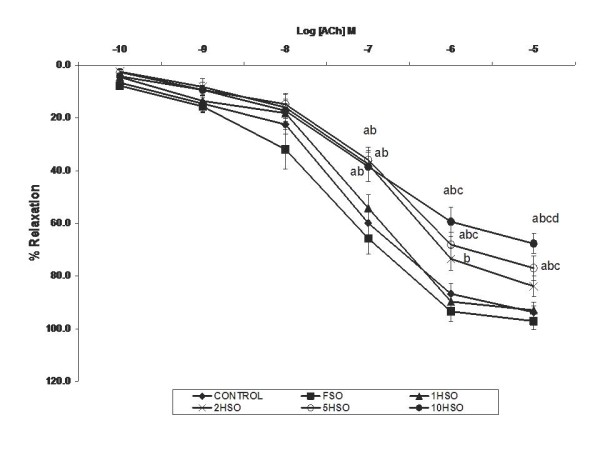
**Effects of fresh and heated soy oil on acetylcholine-induced relaxation in aortic rings.** Endothelium-dependent relaxation in response to acetylcholine (ACh) in aortic rings isolated from rats fed with basal diet (control), fresh soy oil (FSO), soy oil heated once (1HSO), soy oil heated twice (2HSO), soy oil heated five times (5HSO) or soy oil heated ten times (10HSO) at different concentrations. Values are expressed as means ± S.E.M. (n = 7), *p *< 0.05 indicates significant difference compared to ^a^control, ^b^FSO group, ^c^1HSO group, ^d^2HSO group.

**Figure 4:**
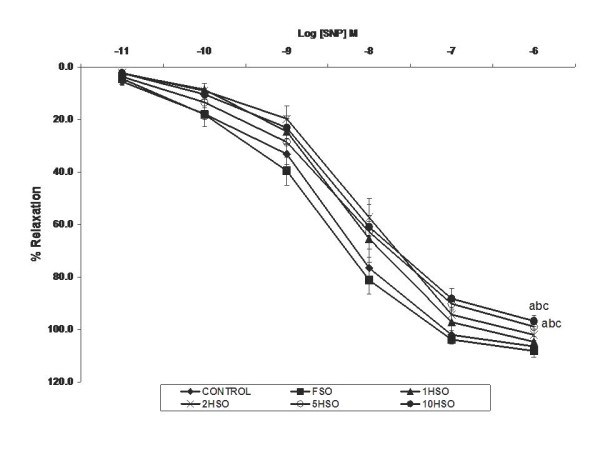
**Effects of fresh and heated soy oil on sodium nitroprusside-induced relaxation in aortic rings. **Endothelium-independent relaxation in response to sodium nitroprusside (SNP) in aortic rings isolated from rats fed with basal diet (control), fresh soy oil (FSO), soy oil heated once (1HSO), soy oil heated twice (2HSO), soy oil heated five times (5HSO) or soy oil heated ten times (10HSO) at different concentrations. Values are expressed as means ± S.E.M. (n = 7), *p *< 0.05 indicates significant difference compared to ^a^control, ^b^FSO group, ^c^1HSO group, ^d^2HSO group.

We observed the effects of fresh and heated soy oil on ACh- and SNP-induced relaxations in aortic rings. Both ACh (Fig. 3) and SNP (Fig. 4) caused concentration-dependent relaxation of contraction induced by PE in aortic rings from all groups of rats. Vasodilator response to ACh was significantly lower (*p *< 0.05) in the 5HSO and 10HSO groups compared to other dietary groups. At the maximal concentration of ACh (10^-5 ^M), 5HSO and 10HSO groups showed a relaxation of 77% and 68% of PE-induced contraction, respectively, compared to the control (94%), FSO (97%), 1HSO (95%) and 2HSO (84%) groups.

In addition, endothelium-independent relaxation induced by SNP at its highest concentration (10^-6 ^M) tested was significantly reduced (*p *< 0.05) in aortic rings obtained from 5HSO (99%) and 10HSO (97%) groups compared to the control (107%), FSO (108%), 1HSO (105%) and 2HSO (102%) groups.

**Figure 5:**
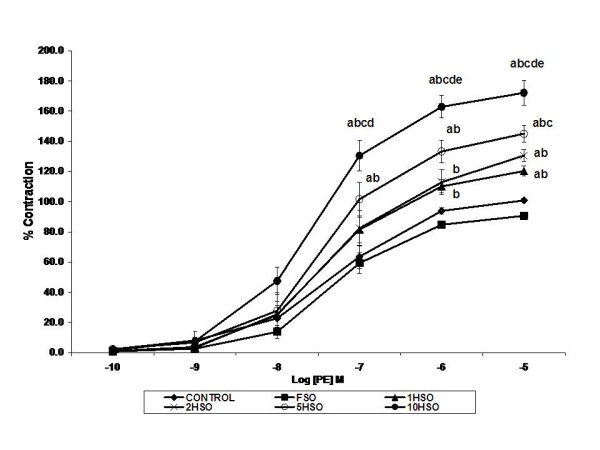
**Effects of fresh and heated soy oil on vascular contraction in aortic rings**. Shown are the contraction induced by phenylephrine (PE) in aortic rings isolated from rats fed with basal diet (control), fresh soy oil (FSO), soy oil heated once (1HSO), soy oil heated twice (2HSO), soy oil heated five times (5HSO) or soy oil heated ten times (10HSO) at different concentrations. Values are expressed as means ± S.E.M. (n = 7), *p *< 0.05 indicates significant difference compared to ^a^control, ^b^FSO group, ^c^1HSO group, ^d^2HSO group, ^e^5HSO group.

We also observed aortic ring contractions in response to increasing concentration of selective α_1_-adrenergic agonist PE. All aortic rings showed a concentration-dependent contraction (Fig. 5). Aortic rings of heated oil groups were susceptible to PE (*p *< 0.05). When tested at PE 10^-5 ^M, a maximum contractile response of 120%, 131%, 145% and 172% of high K^+ ^induced contraction for 1HSO, 2HSO, 5HSO and 10HSO groups, respectively, was recorded. Contraction effects remained similar for both the control (101%) and FSO (91%) groups.

### Plasma heme oxygenase (HO) enzyme concentration

**Figure 6:**
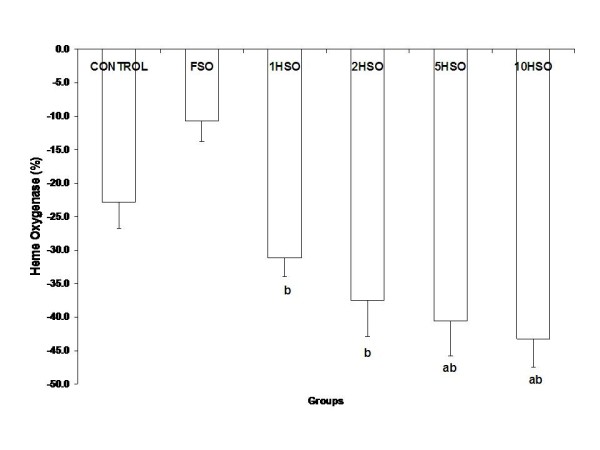
**Effects of fresh and heated soy oil on plasma heme oxygenase level in rats**. Shown are the changes in heme oxygenase level in rats fed with basal diet (control), fresh soy oil (FSO), soy oil heated once (1HSO), soy oil heated twice (2HSO), soy oil heated five times (5HSO) or soy oil heated ten times (10HSO). The results are expressed as percentage based on baseline values. Data are expressed as means ± S.E.M. (n = 7), *p *< 0.05 indicates significant difference compared to ^a^control, ^b^FSO group.

All groups showed a reduction in plasma HO-1 enzyme concentration. Nevertheless, HO-1 level in rats was further decreased significantly (*p *< 0.05) with administration of 10HSO (Fig. 6).

### Plasma angiotensin-converting enzyme (ACE) concentration

**Figure 7:**
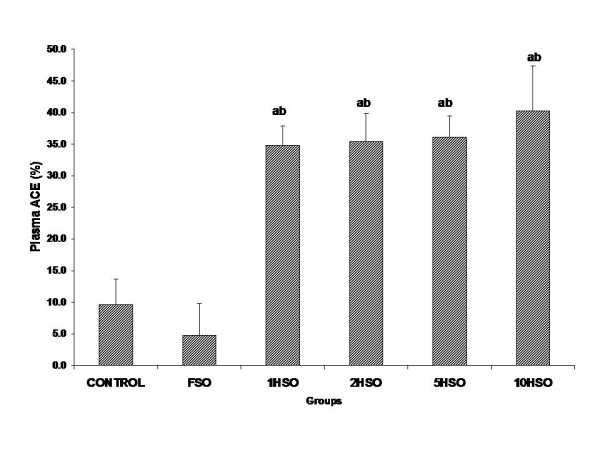
**Effects of fresh and heated soy oil on plasma angiotensin-converting enzyme level in rats**. Shown are the changes in angiotensin-converting enzyme (ACE) level in rats fed with basal diet (control), fresh soy oil (FSO), soy oil heated once (1HSO), soy oil heated twice (2HSO), soy oil heated five times (5HSO) or soy oil heated ten times (10HSO). The results are expressed as percentage based on baseline values. Data are expressed as means ± S.E.M. (n = 7), *p *< 0.05 indicates significant difference compared to ^a^control, ^b^FSO group.

ACE activity of plasma in heated oil groups was significantly higher (*p *< 0.05) than the control and FSO groups (Fig. 7).

## Discussion

The repeated deep-frying process has been documented to be deleterious to the stability of unsaturated fatty acids and other biochemical parameters such as peroxide content, polar material and acid value [32-34] of dietary cooking oil. Fats are usually oxidized by free radicals at the sites of unsaturated bonds in the fatty acid chains. Fats with higher number of unsaturated bonds are prone to oxidation. FSO had a higher ratio of unsaturated fatty acids, thus more susceptible to oxidation (shown in Table 1). Deep-frying oil contained relatively more saturated fatty acids with less unsaturated fatty acids. Peroxide value is usually used as an indicator of the extent of oxidative rancidity. From the results obtained, the extent of oxidation was affected by the number of frying. Repeatedly deep-fried oil also had a rancid odour and darkening of colour compared to the fresh oil.

Incorporation of thermally oxidized oil to the rat's diet was meant to simulate human daily dietary pattern and formed part of the balanced diet. A group fed with fresh oil was included in the study to normalize any effects of the lipid fortification that was not oxidative in origin. There was a significant increase in the body weight at the end of the study for all the groups. This finding suggests that prolonged feeding with fresh or heated soy oil did not affect the growth response. Body weight gained was comparable amongst the control and other test groups. In addition, rats fed with diet enriched with heated soy oil had lower body weight. This may be due to the fatty acid composition in the dietary frying oil. Soy oil is rich in polyunsaturated fatty acid. When the soy oil is repeatedly deep-fried, oxidation degrades oil quality [35], producing sensory changes such as taste, texture and odour that may responsible for the lower food intake in these rats. Additionally, digestibility and absorption of fatty acids might get affected [36].

In present study, rats fed FSO and 1HSO did not show any significant differences in the blood pressure compared to the control. In spite of this, FSO exhibited a tendency to lower blood pressure at the end of experimental period which was in agreement with a recent study performed by Ribeiro Junior *et al*. [37]. Administration of repeatedly heated oil consisting of 2HSO, 5HSO and 10HSO for 24 weeks caused a significant elevation in blood pressure in the rats. This was in accordance with earlier investigations [28,29] demonstrating reheated-palm oil fed group had a significantly greater elevation in blood pressure than the control and fresh-oil fed groups. An earlier study conducted on the cooking oils reported that repeatedly oxidized frying oil is an independent risk factor for hypertension [38].

Our data reported that heated soy oil significantly reduced plasma nitrite levels which are the by-products of NO metabolism. Heated soy oil has been demonstrated in our laboratory to have reduced vitamin E constituents such as α-tocopherol, γ-tocopherol and δ-tocopherol [39] that act as a natural source of antioxidant against generation of free radicals during the frying process. Repeated heating of the cooking oil could not prevent unsaturated fatty acids from oxidative damage through lipid peroxidation. The reduction in nitrite levels could be explained by the enhanced NO sequestration by ROS and inactivation of NO due to the imbalance of antioxidant status. Subsequently, this causes cellular injury and increases blood pressure.

On the contrary, FSO was found to increase nitrites level. FSO contains natural antioxidant which may provide some protective effect by reducing oxidative stress or improving in production of NO [40]. The study by Mahn *et al*. showed that dietary soy protein enhanced expression of nitric oxide synthase (NOS) and antioxidant enzymes [41]. NO production is catalyzed by NOS. It has been documented that a reduction in NO release maybe due to deficiency of NOS [42,43]. Therefore, a decrease in NO production combined with antioxidant/oxidant imbalance may be responsible to the development of endothelial dysfunction [11].

The vascular endothelium may generate a variety of ROS, which under pathological conditions, plays an important contributory role in the pathogenesis of hypertension [44]. ACh was used to study the effects of the heated oil diet on endothelial function. On the other hand, SNP was employed as an endothelium-independent vasodilator in vascular smooth muscle. In the present study, we observed that 5HSO and 10HSO attenuated the endothelium-dependent relaxation induced by ACh as well as the endothelium-independent relaxation induced by SNP in the aortic rings compared to other dietary groups. Alternatively, rats fed with FSO exhibited greater relaxant responses. Diet rich in soy has been reported to have beneficial effect on endothelial function with lower blood pressure [41]. Furthermore, Tousoulis *et al*. were able to show that consumption of soy oil may improve endothelial function in human healthy subjects [45].

Vasorelaxation induced by ACh involves increased bioavailability and release of NO from the endothelium. In contrast, SNP molecules undergo chemical transformation to generate NO which activates cyclic GMP-dependent relaxation in the aortic rings. Heated soy oil attenuated the endothelium-dependent relaxation induced by ACh. However, SNP-induced relaxation was similar in all the groups, indicating the ability of vascular smooth muscle to relax in response to exogenous NO was not impaired in heated oil-fed rats. Heated oil diet selectively impaired endothelium-dependent vasodilatation induced by ACh.

According to the present study findings, it is apparent that heated soy oil diet enhanced PE-induced contraction compared to the control and FSO groups. This indicates an increased in vascular reactivity which would contribute to increasing vascular tone. Generation of free radicals such as superoxide anion has been associated with increased vascular contractile reactivity [46]. This effect may be mediated by reducing NO bioavailability in the aorta of heated oil groups as observed in the present work. In addition, antioxidant protective effect may be diminished when the oil is repeatedly heated.

Measurement of plasma nitrite indirectly indicated there was reduced NO bioavailability either due to reduced NO released from the endothelium or increased inactivation of NO by ROS. Published reports showed that chronic ingestion of repeatedly heated palm oil similarly impaired endothelial *ex-vivo *[29,47]. A limitation in the present work was the absence of measurement of ROS levels to correlate with the endothelial dysfunction.

HO plays a major role in the modulation of blood pressure and vascular tone. It has been postulated that HO-dependent by-products, biliverdin and carbon monoxide have cytoprotective effects against oxidative stress [48,49]. In present experiment, HO level was found to be decreased in all the experimental groups. Nevertheless, repeatedly heated soy oil showed a higher percentage of reduction in plasma HO concentration. Previous research studies reported that over-expression of HO-1 inhibits lipid peroxidation and affects NO metabolism [50,51]. Furthermore, high expression of HO-1 has been linked to an increased in HO enzyme activity and a reduction in blood pressure [52]. In present work, we postulate that chronic consumption of heated soy oil suppressed HO enzyme activity and consequently resulted in unheeded ROS generation.

The present results showed that plasma ACE level was significantly elevated in all heated oil treated groups with 10HSO group showing the highest values. ACE converts inactive Ang I to potent vasoconstrictor, Ang II and raising blood pressure. Ang II increases generation of superoxide free radicals via NADPH/NADH oxidase system. Ang II has a dual role in elevating blood pressure, direct vasoconstrictor effect and increasing production of free radicals which reduces bioavailability of NO and indirectly attenuating endothelium-dependent relaxation responses.

Our results indicated that heated soy oil increased blood pressure and ACE levels with a reduction in NO content. These findings were contradictory to a past study that had reported no influence on blood pressure, ACE activity and an increased in NO concentration [53]. The results could be due to differences in duration of study, the method of oil preparation, the age and strain of the rats. For present study, heated soy oil was fed to adult Sprague-Dawley rats instead of 7-week old spontaneously hypertensive rats and Wistar Kyoto rats [53]. Secondly, the animals in earlier study [53] were fed for 10 weeks compared to our rats which were fed for 24 weeks in present study. In addition, the heating procedure was differed in terms of fried food, duration of frying and cooling of the oil.

Our previous study [29] had used palm oil, with its saturated fatty acid to unsaturated fatty acid ratio close to one compared to soy oil with higher level of polyunsaturated fatty acid. We had found that repeatedly heated palm oil showed a higher percentage of elevation in blood pressure and reduction in nitrite level compare to the control and fresh-oil fed groups. Nevertheless, reheated soy oil showed greater adverse effects as observed in present work. In addition, vasorelaxation in response to ACh was further attenuated with repeatedly heated soy oil compared to palm oil. Previous results from our laboratory showed that consumption of repeatedly heated soy oil and palm oil had caused deterioration in bone histomorphometric properties [54] and lipid peroxidation [55,56] of ovariectomized rats. From these studies, it was concluded that repeatedly heated soy oil worsens the bone histomorphometric changes and increases lipid peroxidation more than the recycled palm oil.

## Conclusion

In conclusion, we suggest that chronic consumption of repeatedly heated soy oil diet leads to endothelial dysfunction. Reheated oil diet promotes oxidative stress resulting in NO sequestration and inactivation. Moreover, repeatedly heated oil causes a significant increased in ACE activity increasing the levels of Ang II with a reduction in HO content, subsequently elevation in blood pressure. Protective effect of fresh soy oil may be lost when the oil is being repeatedly heated. Oxidative stress and endothelial dysfunction are among the critical components in the pathogenesis of hypertension which may be controlled by diet modification. Intake of repeatedly heated soy oil should be restricted due to the harmful implications for health.

## Competing interests

The authors declare that they have no competing interests.

## Authors' contributions

XFL carried out the studies, acquired the data, performed the data analysis, drafted and revised the manuscript. KJ & MRM involved in the design and organization of the study, interpreted the results and revised the manuscript. SD interpreted the results, provided technical assistance in the preparation of the manuscript and revised it. All authors have read and approved the final manuscript.
